# Three approaches to determining clinically meaningful benefit on the Cohen‐Mansfield Agitation Inventory in dementia clinical trials for agitation

**DOI:** 10.1002/trc2.70099

**Published:** 2025-05-15

**Authors:** Kathy Y. Liu, Chineze Ivenso, Rebecca Howard, Penny Rapaport, Suzanne Reeves, Sube Banerjee, Lon S. Schneider, Maria I. Lapid, Agitation MCID Study Group, Robert Howard

**Affiliations:** ^1^ Division of Psychiatry University College London London UK; ^2^ Aneurin Bevan University Health Board St Cadoc's Hospital Newport Wales UK; ^3^ South London and Maudsley NHS Foundation Trust Maudsley Hospital London UK; ^4^ Faculty of Medicine and Health Sciences University of Nottingham Medical School, Queen's Medical Centre Nottingham UK; ^5^ Department of Psychiatry and the Behavioral Sciences, and Department of Neurology Keck School of Medicine, and Leonard Davis School of Gerontology of the University of Southern California Los Angeles California USA; ^6^ Department of Psychiatry & Psychology Mayo Clinic Rochester Minnesota USA

**Keywords:** agitation, Alzheimer, clinically meaningful benefit, dementia, MCID, trial

## Abstract

**INTRODUCTION:**

There is a need to understand the clinical meaningfulness of symptom score changes in treatment trials of dementia‐related agitation. We estimated minimal clinically important differences (MCIDs) for commonly employed agitation scales and contextualized their clinical application.

**METHODS:**

We employed anchor‐ and distribution‐based approaches to determine changes in scores corresponding to minimal symptom improvement. An opinion‐based approach assessed expert clinicians’ agreement on the meaningfulness of score changes through three clinical vignettes.

**RESULTS:**

Minimal symptom improvement for Cohen‐Mansfield Agitation Inventory total score ranged from −4 (over <1 month) to −11 (over 1 to 3 months) points. Greater symptom severity correlated with higher MCID estimates. The clinical importance of score changes was influenced by treatment duration, pharmacological side effects, and impacts on caregiver distress/time resources.

**DISCUSSION:**

The clinical meaningfulness of agitation scale MCIDs is influenced by trial‐specific and clinical factors. Shorter trial durations and measuring caregiver distress/time resources enhance the clinical interpretation of agitation treatment outcomes.

**Highlights:**

- For the CMAI total score, the MCID was −4 points over shorter time scales and −11 points for longer time scales.
- Worse agitation severity was associated with higher MCID estimates.
- There was high expert consensus that a noticeable treatment benefit was not worthwhile if it occurred after 12 weeks or had no impact on caregiver/staff distress/time resources.

## 1 BACKGROUND

Evidence for the efficacy of interventions required for regulatory approval is established by demonstrating statistically significant and clinically meaningful quantitative differences on clinical outcome assessment scales between treatment and placebo arms in randomized controlled trials (RCTs). In May 2023, brexpiprazole was the first drug to be approved by the US Food and Drug Administration for the treatment of agitation in Alzheimer's disease (AD) dementia.^1^ It is widely acknowledged that a difference that is statistically significant is not necessarily clinically meaningful, as statistical significance is largely determined by sample size.^2^ However, there is no consensus on how to interpret the clinical meaningfulness of score changes for agitation trials in AD.

There is no “gold‐standard” method to estimate the minimal clinically important difference (MCID), that is, the smallest score change that is judged to be clinically important. A recommended approach is to combine anchor‐, distribution‐, and/or consensus‐based methods to derive a range of scores within which the MCID falls.^3^, ^4^ Anchor‐based methods link score changes to a subjective judgment of (minimal) change.^4^, ^5^ Distribution‐based methods are statistical approaches based on the variability of scores or score change, typically using measures such as the standard deviation (SD) or effect size to assess magnitude of change.^5^, ^6^ Consensus‐based approaches have included expert opinion collected through, for example, a Delphi survey to determine what constitutes a meaningful change in scores.^7^, ^8^, ^9^ For a distribution‐based approach, MCIDs are usually estimated based on 0.5 × SD of either the baseline or change from baseline mean score.^10^

MCID estimates have been applied to assess the clinical meaningfulness of within‐individual change and between‐group differences, and there are limitations to both approaches.^11^, ^12^ Any single MCID threshold derived from the dichotomization of a continuous symptom scale score will not apply to all individuals and contexts. This is because the clinical relevance of any scale score improvement depends on individual and clinical factors, such as symptom severity and associated risks and costs.^11^, ^13^ It may be more useful to view MCID estimates as representing the smallest mean score change that would be noticeable, defined as a clear, perceptible, and easily communicated change.^13^

Our ability to interpret agitation treatment trial outcomes is limited by insufficient evidence on the clinical meaningfulness of score changes on the most commonly used primary outcome scales employed in these trials. These include the Cohen‐Mansfield Agitation Inventory (CMAI)^14^ or Neuropsychiatric Inventory (NPI).^15^ The CMAI was originally developed for use in nursing homes and assesses the frequency of 29 agitated behaviors over the preceding 2 weeks. The CMAI total score combines the frequency scores for each behavior (ranging from 1 = Never to 7 = Several times an hour), which can be categorized as physically aggressive, verbally aggressive, physically non‐aggressive, or verbally non‐aggressive.^16^ The standard 12‐item NPI assesses the frequency and severity of 12 neuropsychiatric symptoms, or behavioral domains, over the preceding month or other defined period.^17^ It is not specific to agitation but includes an Agitation/Aggression domain. For each domain, a screening question is first asked to determine if the behavior is present. If the answer to this is “Yes,” the domain is scored by multiplying the frequency (1 = Rarely, 2 = Sometimes, 3 = Often, 4 = Very often) and severity (1 = Mild, 2 = Moderate, 3 = Severe) ratings. Earlier studies have reported “MCID” estimates for a CMAI total score of −17 when anchored to *significant* improvement on the Clinical Global Impression‐Change (CGI‐C) from baseline,^18^ or defined clinically meaningful change as −20 points using a triangulation of anchor‐ and distribution‐based methods, approximately corresponding to a two‐category improvement in CGI‐Severity (CGI‐S) and a score of 2 on the CGI‐Improvement (“much improved”).^19^ However, no study has combined MCID estimation approaches to contextualize score changes corresponding to a *minimum* clinically important difference.

This study, therefore, aimed to estimate MCIDs for the CMAI and NPI using anchor‐, distribution‐, and opinion‐based approaches. We also investigated the score change for aggressive versus non‐aggressive symptoms, the specificity of anchored MCID estimates for minimal as opposed to moderate or no change, and the impact of symptom severity on estimates. Using an opinion‐based approach, we aimed to understand factors that influenced clinicians’ agreement on clinical changes that are considered noticeable and worthwhile in the context of specific risks and costs.

## 2 METHODS

To obtain MCID estimates for the CMAI and NPI using anchor‐ and distribution‐based approaches, we used data from two published RCTs^20^, ^21^: (1) a double‐blind, placebo‐controlled 12‐week RCT of risperidone for the treatment of aggression, agitation, and psychosis in older individuals with AD and/or vascular dementia living in nursing homes (*n* = 345) conducted at 14 sites in Australia and New Zealand (RIS‐AUS‐5 study)^20^ and (2) the Study of Mirtazapine for Agitated Behaviors in Dementia (SYMBAD) trial (*n* = 204), a 12‐week, parallel‐group, double‐blind, placebo‐controlled RCT of mirtazapine for the treatment of agitation in AD dementia, conducted in 26 UK centers.^21^ The SYMBAD trial was originally designed as a three‐arm trial, including mirtazapine, carbamazepine, and placebo groups, but the carbamazepine group was closed partway through the trial after 40 people had been randomly allocated to the group. As we included these individuals in the analysis, the total SYMBAD sample in this study was *n* = 244. Eligibility criteria and key summary study sample characteristics for the two trials are described in Methods S1 and S2.

Both RIS‐AUS‐5 and SYMBAD measured agitation using the CMAI, and SYMBAD also used the NPI. We used CMAI frequency scores for analyses and grouped CMAI scores into aggressive and non‐aggressive items, consistent with the RIS‐AUS‐5 study. Of 29 total CMAI items, the 14 aggressive items in the RIS‐AUS‐5 study were hitting, kicking, scratching, grabbing, pushing, hurting self or others, throwing things, cursing or verbal aggression, spitting, tearing things or destroying property, screaming, biting, and making verbal or physical sexual advances. The SYMBAD NPI total score and the NPI agitation/aggression (A/A) (Item 3) domain score were used.

### 2.1 Anchor‐based approach

The anchors in the RIS‐AUS‐5 study were clinician‐ and caregiver‐rated CGI‐S ratings (measured at Visits 1 to 8) and CGI‐C from baseline ratings (measured at Visits 3 to 8). These were encoded as dummy variables (1 to 7 for CGI‐S and −3 to +3 for CGI‐C), where higher values represent worse symptoms. The CMAI scores, ranging from 1 to 7 for each of the 29 items, were recorded at Visit 1 (0 to 7 days before baseline), 2 (baseline), 6 (Week 4), 7 (Week 8), and 8 (Week 12), with higher scores representing more frequent agitation symptoms (1 = Never to 7 = Several times an hour). Thus, for each participant, up to five paired CGI‐S and CMAI measures were available (at Visits 1, 2, 6, 7, and 8), and up to three paired CGI‐S and CMAI score change measures between consecutive visits could be calculated (at Visits 2, 7, and 8). Three paired CGI‐C and CMAI score changes from baseline measures were available at Visits 6, 7, and 8.

The anchor in the SYMBAD study was the final question (Q.32) of the DEMQOL‐Proxy scale,^22^ which asks caregivers to rate the patient's overall quality of life on a four‐point scale (poor, fair, good, very good). As CMAI, NPI, and DEMQOL‐Proxy measures were recorded at up to three visits for each participant (baseline, Week 6, and Week 12), two score change measures between consecutive visits were calculated at Week 6 (change from baseline to Week 6) and 12 (change from Week 6 to Week 12).

Further details on the methods employed for the anchor‐based approach are reported in Method S3, including sensitivity analyses for the RIS‐AUS‐5 anchor‐based analysis.

**RESEARCH IN CONTEXT**

1. **Systematic review**: While earlier studies reported MCID or clinically meaningful change estimates for the CMAI, no study has combined MCID estimation approaches to contextualize score changes corresponding to a *minimum* clinically important difference for agitation scales.
2. **Interpretation**: We found converging MCID estimates using anchor‐, distribution‐, and expert consensus‐based approaches for the CMAI, which were influenced by symptom severity and follow‐up time scales. The smallest detectable improvement for CMAI total score ranged from −4 points (over up to a month) to −11 points (over 1 to 3 months). The clinical importance of score changes was influenced by treatment duration, pharmacological side effects, and impacts on caregiver distress/time resources.
3. **Future directions**: Shorter trial durations and measurement of caregiver distress/time resources will enhance the clinical interpretation of agitation treatment outcomes. Findings have implications for clinical practice, regulatory guidelines, and future research on dementia‐related agitation.

### 2.2 Consensus expert opinion‐based approach

Between June 29 and July 20, 2023, we conducted a survey of dementia experts, who included academic and prescribing or non‐prescribing clinicians with specialist knowledge in the management of agitation in dementia. The survey aimed to assess the agreement on whether described improvements in agitation symptoms within three clinical vignettes were clinically meaningful or not (see Method S4 and Appendices SA and SB). Here, we defined MCID as a noticeable and worthwhile improvement in the context of specific risks and costs.^13^ Basic demographic information (age, race, and sex), occupational background, and prescribing status of participants were collected. Participants were then asked to judge whether the changes in symptoms described in the vignettes represented a noticeable change or not. If so, they were also asked whether the change was worthwhile considering the following: no or small change in caregiver burden; a required duration of treatment of 1 day, 1 week, 4 weeks, or 12 weeks; or (for prescribers) treatment with risperidone 0.5 mg twice a day. The survey comprised three fictional clinical vignettes, each including three scenarios where consecutive improvements in symptoms were described, corresponding to different score changes on CMAI (−2, −4, −6 points) or NPI A/A scales (−4 points) (Table 1).

**TABLE 1:** Design of clinical vignettes and scenarios.

Vignette theme	Setting	Baseline total CMAI score	Change in CMAI scores for scenario	Baseline total NPI A/A score	Change in NPI A/A score	Order of improvements in symptoms (three types/scenarios)
Resistance to care	Residential home	50	−2	12	−4 (severe to moderate)	1. Physical aggression 2. NPI A/A severity 3. Non‐aggressive
Pacing/restlessness	Home	67	−4	12	−4 (severe to moderate)	1. Non‐aggressive 2. NPI A/A severity 3. Verbal aggression
Sexual disinhibition/impulsivity	Residential home	56	−6 or −4	4	NA	1. Verbal aggression (−6) 2. Non‐aggressive (−6) 3. Physical aggression (−4)

*Note*: The resistance to care and pacing/restlessness vignettes included two scenarios describing improvements equivalent to −2 or ‐4 points on CMAI and one scenario describing a −4 improvement on NPI A/A (severity rating). The first two scenarios for the sexual disinhibition/impulsivity vignette described changes equivalent to −6 CMAI points, and the third was equivalent to −4 CMAI points.

Abbreviations: CMAI, Cohen‐Mansfield Agitation Inventory; NPI, Neuropsychiatric Inventory.

The percentage agreement (i.e., proportion of respondents for each question who answered either yes or no) was calculated and compared across scenarios. We classed the degree of agreement as “very high” (≥90%), “high” (75% to 89%), “moderate” (60% to 75%), or “low” (<60%). All analyses were conducted in R version 3.6.3 or 4.3.0.

## 3 RESULTS

### 3.1 RIS‐AUS‐5 data

For participants in the RIS‐AUS‐5 study (*n* = 384), baseline mean CMAI score was 78.7 (SD 22.5) for the total scale, 33.9 (SD 13.0) for aggressive items, and 41.9 (SD 14.2) for non‐aggressive items. Other baseline characteristics are reported in the published study^20^ and shown in Method S2. There was moderate to strong agreement between clinician and carer ratings for the CGI‐S (fixed‐effect regression coefficient 0.73, *p* < 0.0001) and CGI‐C (0.84, *p* < 0.0001) scales.

The CGI‐S anchor showed a moderate correlation with caregiver‐ and clinician‐rated CMAI total scores, which was driven by the presence of aggressive symptoms, reflected in combined and per‐visit Spearman rank correlation coefficients (Table S1). The overall mean CMAI score change corresponding to a one‐category *improvement* in CGI‐S, over 1 or 4 weeks, was around −4 points for total score and around −2 points for aggressive items (Table 2). Sensitivity analyses showed that worse symptom severity, represented by a one‐unit increase in total CMAI score, corresponded to larger MCID estimates for total and aggressive items (Table 2). For clinician ratings, mean total CMAI and aggressive item scores corresponding to a one‐category CGI‐S improvement was significantly different from either no change or a two‐category improvement, but caregiver ratings did not show significant differentiation between a one‐category improvement and no change (Table S2).

**TABLE 2:** MCID estimates anchored to a one‐category improvement on CGI‐S scale and the influence of agitation severity in RIS‐AUS‐5 study.

CGI‐S scale ratings	Simple mean CMAI score change for a one‐category *improvement* (SD)	Effect of one‐unit increase in "earlier" CMAI score on change scores in minimal improvement subgroup	95% CI
Total CMAI frequency score			
Clinician	−4.83 (15.0)	−0.23^**^ ^*^	−0.33 to −0.13
Caregiver	−3.67 (15.2)	−0.19^**^ ^*^	−0.29 to −0.09
CMAI aggressive score			
Clinician	−2.24 (7.9)	−0.28^**^ ^*^	−0.37 to −0.18
Caregiver	−1.66 (7.59)	−0.14^**^	−0.24 to −0.05
CMAI non‐aggressive score			
Clinician	−2.50 (9.9)	−0.22^**^ ^*^	−0.32 to −0.13
Caregiver	−1.81 (9.67)	0.79^**^ ^*^	0.70 to 0.89

*Note*: The mean change in CMAI score corresponding to a one‐category improvement in CGI‐S was calculated using all available measures. The effect of symptom severity, that is, a one‐unit increase in CMAI score, on consecutive change scores was assessed using simple linear regression. Higher CMAI and CGI‐S scores indicate more severe symptoms.

Abbreviations: CMAI, Cohen‐Mansfield Agitation Inventory; CGI‐S, Clinical Global Impression‐Severity; CI, confidence interval.

** *p* < 0.01, ^***^ *p* < 0.001.

The CGI‐C anchor also showed an overall moderate correlation (≥0.3) with caregiver and clinician‐rated CMAI total scores, driven by the presence of aggressive symptoms, but correlations were generally lower compared to the CGI‐S anchor (Table S3). The mean CMAI score change corresponding to “minimal improvement” measured 6, 8, or 12 weeks from baseline was around −11 points for the total score and around −5.5 points for aggressive items (Table 3). Sensitivity analyses showed that worse baseline overall symptom severity corresponded to larger MCID estimates (Table 3). Clinician‐ and caregiver‐rated mean total and aggressive item score change from baseline corresponding to the “Minimal improvement” category was significantly different from the “Moderate improvement” and “No change” categories (Table S2).

**TABLE 3:** Regression coefficients from mixed‐effects models for CGI‐C anchor in RIS‐AUS‐5.

Linear mixed‐effects regression model for the CGI‐C from baseline scale	Mean CMAI score change for "minimal improvement" from baseline	95% CI	One‐unit increase in baseline total CMAI score in minimal improvement subgroup	95% CI
Change in CMAI total frequency score				
Clinician	−10.30^***^	−13.1 to −7.52	−0.30^***^	−0.45 to −0.15
Caregiver	−11.48^***^	−14.3 to −8.70	−0.30^***^	−0.44 to −0.15
Change in CMAI aggressive score				
Clinician	−5.33^***^	−6.83 to −3.83	−0.34^***^	−0.48 to −0.21
Caregiver	−5.95^***^	−7.46 to −4.45	−0.32^***^	−0.44 to −0.20
Change in CMAI non‐aggressive score				
Clinician	−4.42^***^	−6.00 to −2.84	−0.20^**^	−0.34 to 0.07
Caregiver	−4.96^***^	−6.54 to −3.37	−0.18^**^	−0.31 to −0.05

*Note*: The mean change from baseline in CMAI score corresponding to a minimal improvement on the CGI‐C was the estimated marginal mean for this subgroup from linear mixed‐effects regression models. The effect of symptom severity, that is, a 1‐unit increase in baseline CMAI score, on change from baseline scores was assessed using mixed‐effects models. Higher CMAI and CGI‐S scores indicate more severe symptoms.

Abbreviations: CMAI, Cohen‐Mansfield Agitation Inventory; CGI‐C, Clinical Global Impression‐Change; CGI‐S, Clinical Global Impression‐Severity; CI, confidence interval.

* *p* < 0.05, ^**^ *p* < 0.01, ^***^ *p* < 0.001.

### 3.2 SYMBAD data

For the SYMBAD study (*n* = 244), baseline mean CMAI score was 70.4 (SD 17.4) for the total scale, 24.7 (SD 10.1) for aggressive items, and 45.7 (SD 12.9) for non‐aggressive items. The baseline mean NPI total score was 34.9 (SD 19.3). Other baseline characteristics are reported in the published study^21^ and shown in Method S2.

The overall Spearman's rank correlation showed a moderate (≥0.3) correlation only between combined NPI total scores and the DEMQOL‐Proxy Q32 anchor (Table S4). The overall simple mean NPI total score change associated with a one‐category improvement on the DEMQOL‐Proxy overall rating over 6 weeks was −5.72 points (SD 25.3, *n* = 83 observations). The MCID estimates obtained using a distribution‐based approach for both RCT datasets are shown in Table 4.

**TABLE 4:** MCID estimates based on distribution (0.5×SD) of mean baseline or change from baseline scores.

	0.5 × SD mean score	
Scale	Baseline	Change
CMAI (RIS‐AUS‐5)		
Total	11.28	11.35
Aggressive items	6.50	5.98
Non‐aggressive items	7.11	6.55
CMAI (SYMBAD)		
Total	7.62	10.93
Aggressive items	5.03	6.08
Non‐aggressive items	6.43	9.93
NPI (SYMBAD)		
Total	8.92	11.03
Agitation/aggression score	1.14	1.53

*Note*: MCID estimates obtained using a distribution‐based approach for RIS‐AUS‐5 and SYMBAD datasets.

Abbreviations: CMAI, Cohen‐Mansfield Agitation Inventory; MCID, minimal clinically important difference; NPI, Neuropsychiatric Inventory; SD, standard deviation; SYMBAD, Study of Mirtazapine for Agitated Behaviors in Dementia (NCT03031184); RIS‐AUS‐5, A Study on the Effectiveness and Safety of Risperidone in the Treatment of Behavioral Disturbances in Patients with Dementia (NCT00249158)

### 3.3 Consensus expert opinion‐based survey

Most survey respondents were men (56%), White (73%), aged 40 to 59 years (60%), prescribing doctors (82%), working in mental health/psychiatry specialty (89%), and based in the UK (84%) (Table S5). The mean percentage agreement on noticeable change was moderate for a CMAI score change of −2 (67.3%) and −6 (62.3%) and high (82.6%) for a change of −4 (Table 5). There was moderate average agreement (68.2%) that a NPI A/A change from severe to moderate (−4 points) was noticeable. The finding that mean agreement was comparable for −2‐ and −6‐point CMAI score changes suggests that the type and context of the symptom change influenced how noticeable it was judged to be, for example, a 6‐point improvement specific to verbal aggression in the context of sexual disinhibition showed low (50.9%) agreement, whereas a 4‐point improvement specific to verbal aggression in the context of pacing/restlessness showed very high (96.3%) agreement. Mean percentage agreement on whether a change was worthwhile had risperidone 0.5 mg BD been used was low for a CMAI score change of −2 (49.7%), moderate for CMAI score changes of −4 (62.3%) and −6 (61.4%), and moderate for NPI A/A improvement from severe to moderate (62.7%).

**TABLE 5:** Percentage agreement between raters for each survey question.

			Worthwhile change						
			Duration of treatment,^b^ percentage yes				Impact on caregivers/staff ^c^		
Vignette theme	Type of symptom improvement	Noticeable change,^a^ percentage yes	1 day, percentage yes	1 week, percentage yes	4 weeks, percentage yes	12 weeks, percentage yes	No change burden, percentage no	Small change burden, percentage yes	Treatment with risperidone, percentage yes
Resistance to care	1A (Phy −2 CMAI)	69.1	66.7	66.7	27.8	5.6	72.2 (*M* = 2)	81.6	41.5
	1B (NPI −4)	65.5	78.9 (*M* = 7)	90.0 (*M* = 6)	63.2 (*M* = 7)	31.6 (*M* = 7) No	73.5 (*M* = 2)	88.9	55.3
	1C (non‐agg −2 CMAI)	67.3	86.4 (*M* = 5)	91.3 (*M* = 4)	63.6 (*M* = 5)	40.9 (*M* = 5)	51.4 (*M* = 2)	91.9	57.9
Pacing/restlessness	2A (non‐agg −4 CMAI)	69.1	77.3 (*M* = 2)	77.3 (*M* = 2)	45.5 (*M* = 2)	14.3 (*M* = 3) No	69.4 (*M* = 2)	89.5	54.8
	2B (NPI −4)	70.9	81.0 (*M* = 4)	85.7 (*M* = 4)	38.1 (*M* = 4)	23.8 (*M* = 4) No	67.6 (*M* = 2)	87.8	70.0 (*M* = 1)
	2C (verb −4 CMAI)	96.3	77.4	87.1	67.7	35.5 No	52.0 (*M* = 4)	92.3 (*M* = 2)	69.8
Sexually disinhibited/impulsive	3A (verb −6 CMAI)	50.9	69.2 (*M* = 8)	46.2 (*M* = 8)	15.4 (*M* = 8)	7.7 (*M* = 8) No	69.2 (*M* = 2)	85.2 (*M* = 1)	40.6
	3B (non‐agg −6 CMAI)	83.6 (*M* = 1)	85.7 (*M* = 1)	82.1 (*M* = 1)	57.1 (*M* = 1)	28.6 No	57.2 (*M* = 4)	89.1	70.8
	3C (Phy −4 CMAI)	93.9 (*M* = 6)	85.2 (*M* = 2)	89.3 (*M* = 1)	71.4 (*M* = 1)	46.4 (*M* = 1)	38.1 (*M* = 10) Yes	93.5 (*M* = 6)	72.9 (*M* = 6)

*Note*: Answers for the duration of treatment question^b^ are shown for a subgroup (n = 34) after excluding individuals whose answers showed a reversed pattern, which was assumed to be due to misinterpreting the question. The questions are shown below.

Abbreviations: CMAI, Cohen‐Mansfield Agitation Inventory; *M*, number of missing values, non‐agg, non‐aggressive symptom, NPI, NPI severity change from severe to moderate (i.e., patient can be redirected), Phy, physical aggression, verb, verbal aggression.

^a^ Do you consider this to represent a noticeable change in agitation symptoms? (Yes/No) Respondents who answered “yes” to this question were then asked.

^b^ Do you view this change in agitation symptoms to be worthwhile if it only took place after the following duration: 1 day (Yes/No); 1 week (Yes/No); 4 weeks (Yes/No); 12 weeks (Yes/No).

^c^ ‘Do you view this change in agitation symptoms to be worthwhile if it affected caregivers/staff members in the following ways: (i) No changes to their level of personal distress and/or time resources (Yes/No) and (ii) A small reduction in their distress and/or disruption to routine (Yes/No).

^d^ Prescribers (*n* = 46) were asked, “Do you view this change in agitation symptoms to be worthwhile if it had required treatment with 0.5 mg risperidone twice daily? (Yes/No).”

After inspecting the survey responses for the question “Do you view this change in agitation symptoms to be worthwhile if it only took place after the following duration (Yes/No): 1 day; 1 week; 4 weeks; 12 weeks,” it appeared that some participants consistently answered No for shorter durations and Yes for longer durations throughout the survey, which indicated that they misinterpreted the question as asking whether this duration of treatment effect was worthwhile, rather than whether the treatment effect was worthwhile had it taken a certain time to appear. We excluded these participants (*n* = 21) for the subanalysis of this question in Table 5 (the full sample results are in Table S6). In the remaining subsample (*n* = 34), there was moderate to high agreement that improvements after treatment durations of 1 day and 1 week were worthwhile. In contrast, there was low to moderate agreement that treatment durations of at least 4 weeks were worthwhile (with moderate to very high agreement that any change only occurring at 12 weeks of treatment was not worthwhile).

There was low to moderate agreement that any change was worthwhile if this made no difference to caregiver/staff distress and/or time resources. In contrast, there was high to very high agreement that any change making a small difference to caregiver/staff distress and/or time resources would be worthwhile.

In summary, we found that a one‐category improvement on the CGI‐S scale over 1 or 4 weeks corresponded to around −4 points for the CMAI total score and −2 points for CMAI aggressive items. There was high agreement that a CMAI score change of ‐4 was noticeable among surveyed clinicians. Minimal improvement from baseline at 4, 8, or 12 weeks on the CGI‐C scale corresponded to around −11 points for CMAI total and −5.5 points for the aggressive items score, which aligned with distribution‐based findings. A one‐category improvement in overall quality of life on DEMQOL‐Proxy was linked to just under −6 points on the NPI total score.

## 4 DISCUSSION

There was some convergence using anchor‐, distribution‐, and expert consensus‐based approaches to estimate MCIDs for the CMAI. The smallest detectable improvement is likely to be −4 points over shorter time scales (up to a month) and around −11 points for longer time scales (between 1 and 3 months) for the CMAI total score. This may be useful to apply to between‐group differences from agitation RCTs to evaluate the efficacy of drug treatments, as well as to within‐group changes from baseline over the course of the trial. For example, it might be possible to ascertain that at study endpoint, both treatment and placebo groups showed a much larger CMAI change from baseline scores (clearly corresponding to a clinically meaningful improvement) compared to a smaller between‐group mean difference that may not correspond on average to a noticeable change.

The expert clinician survey revealed that the perceived importance of noticeable changes may vary in specific contexts (i.e., after considering risks and costs), which supports this approach to assessing clinical meaningfulness.^13^ For example, there was moderate to very high agreement that any change only occurring after 12 weeks of treatment was not worthwhile. This has implications for agitation treatment RCT durations that last an average of 15 weeks^23^: Shorter trials are needed, including treatments targeting acute agitation symptoms over hours or days.^24^ The survey also showed high to very high agreement that a small improvement in caregiver/staff distress and time resources would make a noticeable change worthwhile, but this outcome is not routinely captured in agitation trials. A final factor is that worse agitation severity may be associated with higher MCID estimates. This is consistent with earlier findings of higher MCID estimates for cognitive and functional scales with greater AD clinical severity.^25^

Our MCID estimate anchored to CGI‐C from baseline was lower than a previously reported “MCID” of −17^18^ and a clinically meaningful change estimate of −20^19^ over a similar timeframe (3 months) also anchored to CGI‐C or CGI‐I and CGI‐S compared to baseline. For the earlier study,^18^ this might be explained by the different methods used to estimate MCID, which compared CGI‐C categories “very much improved”/“improved” and “minimally improved” or “unimproved” using Receiver Operating Characteristic analyses, as well as the study population, which was a longitudinal observational cohort. The correlation between anchor and score change was not reported. The other study^19^ used three RCT datasets to report mean changes in CMAI total score between baseline and Week 12 corresponding to the difference of small improvement versus stable (1‐point decrease vs no change on CGI‐S), or minimally improved versus no change (rating of 3 vs 4 on CGI‐I). The mean CMAI change scores ranged between −10.6 and −13.5, which are more comparable to our findings anchored to CGI‐C. The time difference between measures used to calculate score change may also influence MCID estimates, as the earlier study^18^ found a much smaller MCID of −5 when CGI‐C from baseline was anchored to CMAI scores at 1 month, and the later study^19^ was based on score changes over 3 months. Thus, in addition to the effect of different anchors, length of time between measures of change may also have contributed to the different MCID estimates obtained in this study using CGI‐S (recorded 1 or 4 weeks apart) and CGI‐C (at 4, 8, 12 weeks from baseline) scales in the RIS‐AUS‐5 study.

We found lower correlations with CMAI score using the DEMQOL‐Proxy Q32 anchor compared to CGI‐S or CGI‐C, which may be because overall quality‐of‐life ratings were less specific to agitation symptoms.

### 4.1 Limitations

The CGI‐S scale was not designed to assess *change* in symptoms, and the MCIDs for this anchor were calculated indirectly. As the CGI‐C anchor‐based analyses were based on data obtained at Visits 6, 7, and 8, which were 4, 8, and 12 weeks after baseline, respectively, the findings may be influenced by recall bias, and the longer recall period may explain lower correlations with change in CMAI scores compared to CGI‐S change between consecutive visits. We consider that a one‐category improvement on the CGI‐S and the “minimal improvement” category on the CGI‐C represent the smallest *noticeable* change, but the reliability of clinician‐rated judgements was unmeasured. The clinical vignettes in the survey described symptoms corresponding to CMAI total scores of 50 to 67 points, which was lower than the mean baseline total CMAI scores in the two RCTs (70 and 79 points), and it is possible that higher baseline CMAI scores in the vignettes may have changed the findings. Similarly, only three vignettes were presented, and a larger number of scenarios may improve the reliability of findings. The baseline CMAI scores in the vignettes were nonetheless comparable to the average baseline symptom severity across agitation treatment trials (mean 61.6, SD 14.1, range 47.5to 75.7).^23^ The survey question asking whether clinical improvements were worthwhile if they only occurred after a specific treatment duration was liable to be misinterpreted, and it could have been beneficial to pilot the survey in a small number of prescribers to detect issues and improve the clarity of this question. The cross‐sectional survey was only completed by clinicians, so its findings may not be generalizable to patients or their families, and the stability of responses over time is unknown. As survey respondents were mostly White, doctors, and men working in mental health/psychiatry specialties, a more diverse sample might have altered the findings. We did not record whether survey respondents worked in nursing homes where agitation may be more prevalent or severe, and purposive sampling of these individuals could have improved the content validity of the survey.

Overall, using a combination of anchor‐, distribution‐, and opinion‐based approaches, we found a range of MCID estimates for agitation scale scores that may be context‐dependent. The size of noticeable score change is influenced by the methods used to derive MCID estimates, the severity of agitation symptoms, and potentially the type and context of symptoms. The importance of these changes is influenced by the personal/clinical consideration of associated risks and costs. Establishing a worthwhile treatment benefit from trial findings may be more difficult with longer treatment durations and when the impact on caregiver/staff distress/time resources is not fully captured. Our findings are relevant for regulatory guidelines for the conduct and approval of trials on dementia‐related agitation, as well as helping to guide clinical treatment decisions and setting realistic expectations for patient outcomes. Future research should explore the variability and stability of MCID estimates across different populations and settings and incorporate the needs and preferences of people with dementia and their caregivers.

## AGITATION MCID STUDY GROUP

Alan Thomas PhD, Alison Young MSc, Andrew Sommerlad PhD, Anitha Howard MRCPsych, Aparna Mordekar MRCPsych, Becca Stilwell DClinPsy, Brid Kerrigan FRCPsych, Cherry Shute, Claire Pocklington MRCPsych MSc, Claire Potter MRCPsych, Constantine Lyketsos MD MHS, Geir Selbæk MD PhD, Gemma Forshaw DClinPsy, Gill Livingston MD, Gregor Russell MD, Jonathan Huntley PhD, Joseph P.M. Kane MRCPsych, Leah Clatworthy DClinPsy, Leo Tumelty MB BCh, Leonidas Chouliaras PhD, Malgorzata Raczek MRCPsych PhD, Manoj Rajagopal MRCPsych MSc, Marie‐Andrée Bruneau MD MSc FRCPC, Martin Smallbrugge PhD MD, Naaheed Mukadam PhD, Oli Sparasci BMBS Mres BSc, Patrick Chance MBChB MRCPsych, Paul C. Donaghy PhD, Petr Zvolsky, Pierre N Tariot MD, Raymond T.C.M. Koopmans MD PhD, Roinin McNally MRCPsych, Sarah Wilson MRCPsych MSc, Sudip Sikdar FRCPsych, MD, Terence W.H. Chong FRANZP, Tom Dening FRCPsych, Vivek Pattan MRCPsych.

## CONFLICT OF INTEREST STATEMENT

M.L. was supported in part by Grant R01AG061100 from the National Institute on Aging and has no financial relationships to disclose. L.S. and C.I. declare sources of funding in their International Committee of Medical Journal Editors (ICJME) Disclosure of Interest Form. Other authors report no financial relationships with commercial interests. Author disclosures are available in the supporting information.

## CONSENT STATEMENT

Written informed consent to take part in the trials was obtained from participants or their legal representative.

## Supporting information



Supporting Information

Supporting Information
