# Supplementary material for: Three approaches to determining clinically meaningful benefit on the Cohen‐Mansfield Agitation Inventory in dementia clinical trials for agitation
Source: Alzheimers Dement (N Y). 2025 May 15;11(2):e70099. doi: 10.1002/trc2.70099 (PMC12079343; doi:10.1002/trc2.70099)
Supplement: Supplementary file 2 — Supporting Information [file TRC2-11-e70099-s002.docx]

SUPPLEMENTARY FILES

[**Methods S1.** 2](#_Toc192770597)

[**Methods S2.** 3](#_Toc192770598)

[**Methods S3** 4](#_Toc192770599)

[**Methods S4.** 7](#_Toc192770600)

[**Table S1. Spearman’s rank correlation coefficient for the CGI-S anchor by visit.** 9](#_Toc192770601)

[**Table S2 . Comparison of CMAI score change for minimal improvement to score change for no change or moderate improvement on the CGI-S/C scales.** 10](#_Toc192770602)

[**Table S3. Spearman’s rank correlation coefficient for the CGI-C anchor by visit.** 11](#_Toc192770603)

[**Table S4. Spearman’s rank correlation coefficient for the DEMQOL-Proxy Q.32 anchor by visit.** 12](#_Toc192770604)

[**Table S5. Characteristics of clinicians who completed the online survey.** 13](#_Toc192770605)

[**Table S6. Percentage agreement on duration of treatment for the whole survey sample.** 14](#_Toc192770606)

[**Appendix A. Email sent to identified dementia experts.** 15](#_Toc192770607)

[**Appendix B. Clinical vignettes and scenarios.** 18](#_Toc192770608)

###

### **Methods S1.**

Eligibility criteria of trials:

| Trial | Inclusion criteria | Exclusion criteria |
| --- | --- | --- |
| RIS-AUS-5 [1] | - Diagnosis of dementia (Alzheimer’s type, vascular dementia, or combination of the two, i.e. mixed dementia), according to DSM-IV. - ≥55 years old - Score ≥4 on Functional Assessment Staging Test - Score ≤23 on MMSE - Resided in nursing home for at least 1 month prior to enrollment. - Aggressive behaviours, measured using CMAI, defined as scoring ≥ 4 on at least 1 aggressive item, or a score of 3 on at least 2 aggressive items, or a score of 2 on at least 3 aggressive items, or 2 aggressive items occurring at a frequency of 2 and 1 at a frequency of 3. | - Medical or neurological conditions other than dementia that diminish cognitive function, other types of dementia, major depression within the last 6 months, other psychiatric disorders that could have accounted for observed psychotic disturbances, a history of tardive dyskinesia, clinically uncontrolled organic disease, clinically relevant laboratory abnormalities, administration of a depot neuroleptic within 2 treatment cycles, a history of neuroleptic malignant syndrome or an allergic reaction to neuroleptic drugs, history of failure to respond to risperidone treatment of at least 4 weeks’ duration, and participation in clinical trial(s) with any investigational drugs during the 4 weeks preceding selection. |
| SYMBAD [2] | - Met NINCDS-ADRDA Alzheimer’s criteria for probable or possible Alzheimer’s disease, ascertained by referring psychiatrists - Coexisting agitation, defined as CMAI score of 45 or more. - Evidence that the aetiology of agitated behaviours had been investigated and not responded to non-pharmacological management according to the Alzheimer’s Society and Department of Health algorithm. | - Considered clinically too critically unwell for participation (e.g. suicide risk), had absolute contraindications to trial drugs (hypersensitivity to mirtazapine or carbamazepine, or structurally related drugs, second-degree atrioventricular block, use of monoamine oxidase inhibitors, or a history of bone marrow depression or hepatic porphyria), were already taking antidepressants or antipsychotics, were in another Investigational Medicinal Product trial, were women under the age of 55 of childbearing potential, or had no family or professional carer informant available. |

### **Methods S2.**

Key summary baseline characteristics of trial participants for RIS-AUS-5 [1]:

| Characteristic | Placebo (N=156) | Risperidone (N=153) |
| --- | --- | --- |
| Female, N (%) | 113 (72.4) | 109 (71.2) |
| Age in years, mean (SE) | 82.7 (0.64) | 83.2 (0.51) |
| Diagnosis, N (%) |  |  |
| Alzheimer’s dementia | 93 (59.6) | 87 (56.9) |
| Vascular dementia | 44 (28.2) | 44 (28.8) |
| Mixed dementia | 19 (12.2) | 22 (14.4) |
| MMSE score, mean (SE) | 5.78 (0.46) | 5.14 (0.45) |
| CMAI total aggression (SE) | 33.0 (0.99) | 34.1 (1.05) |

Key summary baseline characteristics of trial participants for SYMBAD [2]:

| Characteristic | Placebo (N=102) | Mirtazapine (N=102) | Carbamazepine (N=40) |
| --- | --- | --- | --- |
| Female, N (%) | 59 (58) | 76 (75) | 32 (80) |
| Age in years, mean (SD) | 82.8 (7.7) | 82.2 (7.8) | 83.2 (8.1) |
| MMSE score, mean (SD) | 16.1 (6.7) *n=50* | 13.4 (8.1) *n=52* | 12.0 (6.0) *n=23* |
| CMAI total score (SD) | 69.8 (17.1) | 71.1 (16.4) | 70.0 (21.0) |
| NPI total score (SD) | 34.9 (18.2) | 32.7 (16.7) *n=98* | 40.5 (26.1) |
| NPI agitation/aggression subscore (SD) | 5.6 (3.4) | 5.6 (3.2) *n=99* | 6.5 (4.0) |

### **Methods S3**

*S3.1 Anchor-based approach*

As a moderate correlation of ≥0.3 between anchor and a clinical outcome assessment (COA) scale has been recommended for the appropriate estimation of an MCID [3], we calculated the combined and per visit Spearman’s rank correlation coefficient, which assesses the cross-sectional monotonic relationship between two ordinal/continuous variables, for the following anchor-based MCID estimates: 1) between CMAI and CGI-S (RIS-AUS-5), 2) CMAI change and CGI-C from baseline (RIS-AUS-5), 3) CMAI and DEMQOL-Proxy Q.32 (SYMBAD), and 4) NPI and DEMQOL-Proxy Q.32 (SYMBAD).

We then estimated the change in CMAI/NPI score corresponding to a minimal improvement, defined as, 1) a 1-category improvement on the CGI-S scale, 2) ‘Minimal improvement’ from baseline category on the CGI-C scale, and 3) a 1-category improvement on Q.32 of DEMQOL-Proxy. Earlier studies of MCIDs in AD have used similar approaches. A 1-point worsening from any category on the Global Deterioration Scale and the ‘minimal worsening’ category of the CGI-C from baseline ratings served as clinician-rated anchors for minimal worsening on cognitive scales in individuals with mild cognitive impairment [4]. The DEMQOL-Proxy was previously used as an anchor to investigate the MCID for the Sleep Disorders Inventory in individuals with dementia, although only the correlation between changes in total score was reported [5]. For RIS-AUS-5 data, which included at least two within-individual CMAI score change measures, CMAI change from baseline in a model that allowed different intercepts for each participant showed a better fit than one that assumed a single overall mean change in total score without accounting for grouping effects, based on the Akaike Information Criterion (AIC) (see Table for Methods S3.1). Thus, linear mixed-effects regression models were used for anchor-based MCID estimations between CMAI change and CGI-C from baseline scores (but not CMAI and CGI-S change scores between consecutive visits). For the CGI-C anchor, a binary indicator variable was created to distinguish individuals who experienced a minimal improvement versus those who did not, and the estimated marginal means from a linear mixed-effects model was obtained to derive the mean CMAI change score (adjusted for random effects) for the minimal improvement group in the model. MCID estimates for the CGI-S and DEMQOL-Proxy Q.32 anchors were calculated using the simple overall mean change in score corresponding to a one-unit improvement on the scales using all available measures.

**Table for Methods S3.1. AIC differences between simple and random intercepts models**

| Outcome ratings | AIC 1 | AIC 2 | △AIC |
| --- | --- | --- | --- |
| Raw CMAI total score | 14462 | 13920 | -542 |
| CMAI total score change between consecutive visits | 7626 | 7627 | 1 |
| CMAI total score change from baseline | 7870 | 7575 | -295 |

For analyses of RIS-AUS-5 data involving ≥2 repeated CMAI score change measures within individuals, the AIC differences between a model that assumed a single overall mean (change) in total score without accounting for grouping effects (AIC 1), and a model that allowed different intercepts for each participant (AIC 2) were calculated, with lower values indicating better model fit.

*S3.2 Sensitivity analyses for RIS-AUS-5 anchor-based approach*

For RIS-AUS-5 data, we assessed the specificity of minimal change for the CGI-S/C anchors by testing whether this was significantly different to the mean CMAI score change corresponding to either no change (a 0-category change on the CGI-S or the ‘Unchanged’ category on the CGI-C) or moderate improvement (a 2-category improvement on the CGI-S or the ‘Moderate improvement’ category on the CGI-C). This was achieved through analysis of variance (ANOVA) for the CGI-S anchor and pairwise comparisons on obtained estimated marginal means for each group category in a linear mixed-effects model for the CGI-C anchor. We also assessed the influence of symptom severity on MCID estimates by including a measure of severity (for CGI-S, the earlier CMAI score in the pair of scores used to calculate CMAI differences, and for CGI-C, the baseline CMAI score) as a covariate in simple (CGI-S) or mixed (CGI-C) effects models for the subgroup who experienced a minimal improvement. We also assessed the average agreement between carer and clinician CGI-S or CGI-C scores via the fixed effect regression coefficient from a linear mixed-effects model that included both variables.

### **Methods S4.**

A survey development group (authors KL, CI, ReH, PR, SR, RoH) discussed the design and content and with author LS identified the following groups of clinical experts (sample size where known): Royal College of Psychiatrists Old Age Faculty in England (*JiscMail* mailing list, n=177), Wales, Scotland and Northern Ireland, the National Steering Group for Dementia in Wales, the Division of Clinical Psychology Faculty of the Psychology of Older People, the National Institute for Health Research dementia specialty leads (n=17), International Psychogeriatric Association 2016 meeting registrants (n=48), and individual clinicians/colleagues (n=19).

The link to the survey in Microsoft Forms was included in an email (see Supplementary Appendices A and B in the online supplement) sent directly to identified experts, who were offered group/consortium authorship on any publication and a certificate of participation. Ethical approval was obtained from London - Queen Square Research Ethics Committee (ref: 19/LO/1825).

### **Table S1. Spearman’s rank correlation coefficient for the CGI-S anchor by visit.**

| Visit | CGI-S rater | Spearman’s rank correlation coefficient | | | Simple mean CMAI score change associated with a one-category improvement (SD) | | |
| --- | --- | --- | --- | --- | --- | --- | --- |
|  |  | CMAI total frequency score | Aggressive items | Non-aggressive items | Total | Aggressive items | Non-aggressive items |
| 1 | Clinician | 0.29*** | 0.42*** | 0.07* |  |  |  |
|  | Caregiver | 0.36*** | 0.51*** | 0.11* |  |  |  |
| 2 | Clinician | 0.38*** | 0.44*** | 0.20*** | -3.61 (15.3) | -2.80 (8.31) | -0.61 (9.93) |
|  | Caregiver | 0.47*** | 0.54*** | 0.23*** | -0.14 (14.2) | -0.59 (7.94) | -0.74 (8.90) |
| 6 | Clinician | 0.41*** | 0.55*** | 0.23*** |  |  |  |
|  | Caregiver | 0.46*** | 0.58*** | 0.27*** |  |  |  |
| 7 | Clinician | 0.48*** | 0.52*** | 0.30*** | -7.18 (14.2) | -2.65 (7.71) | -4.14 (8.38) |
|  | Caregiver | 0.54*** | 0.63*** | 0.32*** | -8.70 (12.8) | -2.95 (6.58) | -5.54 (8.38) |
| 8 | Clinician | 0.56*** | 0.58*** | 0.42*** | -3.65 (15.4) | -1.42 (7.81) | -2.49 (10.9) |
|  | Caregiver | 0.60*** | 0.65*** | 0.43*** | -2.46 (17.3) | -1.53 (8.07) | -0.83 (10.7) |
| All visits combined | Clinician | 0.46*** | 0.53*** | 0.28*** | -4.83 (15.0) | -2.24 (7.9) | -2.50 (9.9) |
|  | Caregiver | 0.53*** | 0.61*** | 0.31*** | -3.67 (15.2) | -1.66 (7.59) | -1.81 (9.67) |

***p<0.001, *p<0.05

### **Table S2. Comparison of CMAI score change for minimal improvement to score change for no change or moderate improvement on the CGI-S/C scales.**

| Anchor | | Difference between minimal improvement and no change | Difference between minimal and moderate improvement |
| --- | --- | --- | --- |
|  |  | Estimate | Estimate |
| CGI-S total | Clinician | -4.10* | 6.80** |
|  | Caregiver | -2.25 | 9.20*** |
| CGI-S aggressive items | Clinician | -2.15** | 3.84** |
|  | Caregiver | -1.03 | 4.48*** |
| CGI-S non-aggressive items | Clinician | -1.81 | 2.64 |
|  | Caregiver | 0.87 | 4.53** |
| CGI-C total | Clinician | -4.83** | 6.26** |
|  | Caregiver | -6.79*** | 6.12** |
| CGI-C aggressive items | Clinician | -2.25* | 3.74*** |
|  | Caregiver | -3.65*** | 3.54*** |
| CGI-C non-aggressive items | Clinician | -2.11 | 2.39* |
|  | Caregiver | -2.69** | 2.51* |

For the CGI-S anchor, analysis of variance and post-hoc Tukey tests were used to compare mean CMAI score change between minimal, moderate, or no improvement groups. For the CGI-C anchor, pairwise comparisons were conducted between estimated marginal means of CMAI score change for each group obtained from mixed effects models.

*p<0.05, **p<0.01 *** p < 0.001

###

### **Table S3. Spearman’s rank correlation coefficient for the CGI-C anchor by visit.**

| Visit | CGI-C rater | Spearman’s rank correlation coefficient | | |
| --- | --- | --- | --- | --- |
|  |  | CMAI total change from baseline | Aggressive items | Non-aggressive items |
| 6 | Clinician | -0.27*** | -0.29*** | -0.17** |
|  | Caregiver | -0.36*** | -0.35*** | -0.28*** |
| 7 | Clinician | -0.30*** | -0.29*** | -0.23*** |
|  | Caregiver | -0.40*** | -0.35*** | -0.33*** |
| 8 | Clinician | -0.44*** | -0.43*** | -0.35*** |
|  | Caregiver | -0.39*** | -0.42*** | -0.30*** |
| All visits combined | Clinician | -0.35*** | -0.35*** | -0.27*** |
|  | Caregiver | -0.39*** | -0.38*** | -0.31*** |

Combined and per visit Spearman’s rank correlations between CMAI scores and clinician- and caregiver- rated CGI-C from baseline ratings. The combined visits correlation between CGI-C and CMAI total and aggressive items change from baseline fulfilled the recommended correlation of ⩾0.3 for the appropriate estimation of an MCID.

*** p < 0.001 ** p<0.01

### **Table S4. Spearman’s rank correlation coefficient for the DEMQOL-Proxy Q.32 anchor by visit.**

| Visit | Spearman’s rank correlation coefficient | | | | |
| --- | --- | --- | --- | --- | --- |
|  | CMAI total | CMAI aggressive items | CMAI non-aggressive items | NPI total score | NPI agitation/ aggression |
| 1 | -0.15* | -0.21** | -0.08 | -0.14* | -0.06 |
| 2 | -0.32*** | -0.28*** | -0.29*** | -0.24** | -0.22* |
| 3 | -0.33*** | -0.30*** | -0.27*** | -0.10 | -0.19* |
| All visits combined | -0.28*** | -0.26*** | -0.22*** | -0.36*** | -0.28*** |

Combined and per visit Spearman’s rank correlations between CMAI scores and clinician- and caregiver- rated CGI-C ratings. Only the combined visits correlation between DEMQOL-Proxy Q.32 and NPI total score fulfilled the recommended correlation of ⩾0.3 for the appropriate estimation of an MCID.

*** p < 0.001 ** p<0.01 * p<0.05

### **Table S5. Characteristics of clinicians who completed the online survey.**

| Characteristic | Frequency |
| --- | --- |
| Clinical profession |  |
| Doctor | 45 |
| Psychologist | 2 |
| Nurse | 1 |
| Other | 7 |
| Specialty |  |
| Mental health or psychiatry | 49 |
| Elderly care or geriatric medicine | 4 |
| Neurology | 1 |
| Other | 1 |
| Country of work |  |
| United Kingdom | 46 |
| United States | 4 |
| Netherlands | 2 |
| Canada | 1 |
| Australia | 1 |
| Norway | 1 |
| Gender identity |  |
| Man | 31 |
| Woman | 24 |
| Ethnicity |  |
| White | 40 |
| Asian, Asian British | 11 |
| African or Caribbean, Black British | 2 |
| Mixed or multiple ethnic groups | 2 |
| Age in years |  |
| 40-59 | 33 |
| 20-39 | 13 |
| 60+ | 9 |

###

### **Table S6. Percentage agreement on duration of treatment for the whole survey sample.**

| Vignette | Question | Worthwhile ^a^? %Yes | | | |
| --- | --- | --- | --- | --- | --- |
|  |  | 1 day | 1 week | 4 weeks | 12 weeks |
| Resistance to care | 1A (Phy) | 35.3 | 65.7 | 51.4 | 22.9 |
|  | 1B (NPI) | 46.9 | 87.9 | 73.5 | 39.4 |
|  | 1C (non-agg) | 59.4 | 91.2 | 71.4 | 42.4 |
| Pacing/restless | 2A (non-agg) | 52.9 | 83.3 | 59.5 | 26.5 |
|  | 2B (NPI) | 58.8 | 86.1 | 54.1 | 34.3 |
|  | 2C (verb) | 61.7 | 89.8 | 72.5 | 41.7 |
| Sexually disinhibited/impulsive | 3A (verb) | 52.0 | 64.3 | 42.3 | 28.0 |
|  | 3B (non-agg) | 69.0 | 82.2 | 68.2 | 40.5 |
|  | 3C (Phy) | 68.3 | 88.6 | 77.8 | 52.4 |

^a^ To respondents who judged that the described change was noticeable, the next question asked was ‘Do you view this change in agitation symptoms to be worthwhile if it only took place after the following duration: 1 day (Yes/No), 1 week (Yes/No), 4 weeks (Yes/No), 12 weeks (Yes/No)’.

We classed the degree of agreement as ‘very high’ (⩾90%), ‘high’ (75-89%), ‘moderate’ (60-75%) or ‘low’ (<60%).

Abbreviations: Phy = physical aggression, non-agg=non-aggressive symptom, verb=verbal aggression.

###

### **Appendix A. Email sent to identified dementia experts.**

Dear Colleague,

What is a clinically meaningful improvement in agitation symptoms in individuals with dementia?

We are interested in determining what experienced dementia clinicians would regard as the minimum worthwhile improvement in agitation that might be seen with treatment.

We would like to invite you to participate in an online Delphi study to investigate the clinical relevance of small changes on agitation scale scores. The study is designed to help us to interpret the results of recent and future drug treatment trials of agitation in Alzheimer's disease.

The study starts with this online [survey](https://eur01.safelinks.protection.outlook.com/?url=https%3A%2F%2Fscanmail.trustwave.com%2F%3Fc%3D261%26d%3D27ic5MStRkhDrZrRNjxZbIjVew-ohh9H40YZW2pbLw%26u%3Dhttps%253a%252f%252fforms.microsoft.com%252fe%252fneyCKuUfGF&data=05%7C01%7Ckathy.liu%40ucl.ac.uk%7C4a1a05c9fb024d59121a08db78912b20%7C1faf88fea9984c5b93c9210a11d9a5c2%7C0%7C0%7C638236337122148431%7CUnknown%7CTWFpbGZsb3d8eyJWIjoiMC4wLjAwMDAiLCJQIjoiV2luMzIiLCJBTiI6Ik1haWwiLCJXVCI6Mn0%3D%7C3000%7C%7C%7C&sdata=95SHWAyr0ZrXfooZk5AvtHtmDb%2BdfvFy%2FDOig8IncHk%3D&reserved=0) which takes around 15 minutes to complete. We would be very grateful if this could be submitted within one week.

All participants will be offered **group/consortium authorship** when the study is accepted for publication. We can also provide a certificate for participation in a research study to improve the quality of clinical interventions and services, which can be used for appraisals.

The full study information for participants that met requirements for ethical approval is provided below.

Thank you for your consideration.

Dr Kathy Liu

Old Age Psychiatry specialty registrar (ST6) and MRC Clinical Research Training Fellow

Division of Psychiatry, University College London

Email: kathy.liu@ucl.ac.uk

-------------------------------------------------------

Information about this research

We are inviting you to take part in a Delphi survey to establish clinical consensus on the minimal clinically important difference (MCID) in score for two commonly used agitation scales, the Cohen Mansfield Agitation Inventory (CMAI) and the Neuropsychiatric Inventory (NPI). The findings will contribute to a wider investigation of the minimal clinically important difference (MCID) of agitation scale scores in Alzheimer's disease.

You are being invited to take part as you have been identified as an expert in this area, as you are an academic and/or a clinician who is involved in the assessment and/or management of people with dementia-related agitation.

The study is being run by Dr Kathy Liu at University College London, as part of her PhD project on the role of the noradrenergic system in agitation in Alzheimer’s disease. The study team members include Dr Rebecca Howard, Prof. Robert Howard, Dr Chineze Ivenso, Dr Penny Rapaport, Prof. Suzanne Reeves, and Prof. Lon Schneider.

The findings will help us to interpret the results of drug treatment trials of agitation in Alzheimer’s disease.

What does taking part involve?

A Delphi survey aims to obtain a consensus opinion from a group of experts.

We would like you to complete an electronic questionnaire. You will be asked about your occupational background, including whether you prescribe medications, and some basic demographic information.

After reading three scenarios each stemming from three fictional case vignettes, you will be then asked to give your clinical opinion on whether a described improvement in agitation symptoms represents a clinically meaningful change.

The survey should take around 15 minutes to complete, and we would like you to complete this within one week.

The link to the survey is [https://forms.microsoft.com/e/neyCKuUfGF](https://eur01.safelinks.protection.outlook.com/?url=https%3A%2F%2Fscanmail.trustwave.com%2F%3Fc%3D261%26d%3D27ic5MStRkhDrZrRNjxZbIjVew-ohh9H40YZW2pbLw%26u%3Dhttps%253a%252f%252fforms.microsoft.com%252fe%252fneyCKuUfGF&data=05%7C01%7Ckathy.liu%40ucl.ac.uk%7C4a1a05c9fb024d59121a08db78912b20%7C1faf88fea9984c5b93c9210a11d9a5c2%7C0%7C0%7C638236337122148431%7CUnknown%7CTWFpbGZsb3d8eyJWIjoiMC4wLjAwMDAiLCJQIjoiV2luMzIiLCJBTiI6Ik1haWwiLCJXVCI6Mn0%3D%7C3000%7C%7C%7C&sdata=95SHWAyr0ZrXfooZk5AvtHtmDb%2BdfvFy%2FDOig8IncHk%3D&reserved=0)

After the first round of responses, we will send a summary of responses to the clinical questions, collated anonymously, in an additional questionnaire, and this process is repeated until adequate consensus is reached. We anticipate that you will be emailed no more than three times.

The study may not benefit you directly but is intended to benefit research in treatments for agitation in Alzheimer’s disease. We do not anticipate any risks of taking part.

What happens if I do not want to take part?

If you prefer not to take part, you can simply ignore this email.

Confidentiality and dissemination of results

The responses will be treated confidentially and collated anonymously, and the *collated* results will be disseminated to other participants in the Delphi study.

The results of the study will be published in a peer-reviewed journal.

*How will we use information about you?*

- We will need to use information from you for this research project.
- This information will include your name, email address, characteristics (professional role and specialty, country of work, gender, age grouping and ethnicity), as well as your answers to the main survey. People on the study will use this information to do the research or to check records to make sure that the research is being done properly.
- People who do not need to know who you are will not be able to see your name or contact details. Your data will have a code number instead.
- We will keep all information about you safe and secure.
- Once we have finished the study, we will keep some of the data so we can check the results. We will write our reports in a way that no-one can work out that you took part in the study.

*What are your choices about how your information is used?*

- You can stop being part of the study at any time, without giving a reason, but we will keep information about you that we already have.
- We need to manage your records in specific ways for the research to be reliable. This means that we won’t be able to let you see or change the data we hold about you.

*Where can you find out more about how your information is used?*

You can find out more about how we use your information

- at [www.hra.nhs.uk/information-about-patients/](https://eur01.safelinks.protection.outlook.com/?url=https%3A%2F%2Fscanmail.trustwave.com%2F%3Fc%3D261%26d%3D27ic5MStRkhDrZrRNjxZbIjVew-ohh9H4xVOAzlcfQ%26u%3Dhttps%253a%252f%252furldefense.com%252fv3%252f__https%253a%252fwww.hra.nhs.uk%252finformation-about-patients%252f__%253b%2521%2521LIr3w8kk_Xxm%2521t5EZwZgJUPTvH5LzXuPI8SJangF5sNrjK0UWe8zNTM0WP-NzPJ8-oS-Cwd1onYMONDScVEn3PwA2oK7GwFJv%2524&data=05%7C01%7Ckathy.liu%40ucl.ac.uk%7C4a1a05c9fb024d59121a08db78912b20%7C1faf88fea9984c5b93c9210a11d9a5c2%7C0%7C0%7C638236337122148431%7CUnknown%7CTWFpbGZsb3d8eyJWIjoiMC4wLjAwMDAiLCJQIjoiV2luMzIiLCJBTiI6Ik1haWwiLCJXVCI6Mn0%3D%7C3000%7C%7C%7C&sdata=hnBrThtNI4S7s5%2FPylSChfx76l9yiexCX1QKSwPHw5k%3D&reserved=0)
- and at [www.hra.nhs.uk/patientdataandresearch](https://eur01.safelinks.protection.outlook.com/?url=https%3A%2F%2Fscanmail.trustwave.com%2F%3Fc%3D261%26d%3D27ic5MStRkhDrZrRNjxZbIjVew-ohh9H4xcZUW5beg%26u%3Dhttps%253a%252f%252furldefense.com%252fv3%252f__http%253a%252fwww.hra.nhs.uk%252fpatientdataandresearch__%253b%2521%2521LIr3w8kk_Xxm%2521t5EZwZgJUPTvH5LzXuPI8SJangF5sNrjK0UWe8zNTM0WP-NzPJ8-oS-Cwd1onYMONDScVEn3PwA2oFL_7kPq%2524&data=05%7C01%7Ckathy.liu%40ucl.ac.uk%7C4a1a05c9fb024d59121a08db78912b20%7C1faf88fea9984c5b93c9210a11d9a5c2%7C0%7C0%7C638236337122148431%7CUnknown%7CTWFpbGZsb3d8eyJWIjoiMC4wLjAwMDAiLCJQIjoiV2luMzIiLCJBTiI6Ik1haWwiLCJXVCI6Mn0%3D%7C3000%7C%7C%7C&sdata=bPgSmIpRS51r36Dpj%2BQkNW4lzg7L5BFkK1yYU8RRfpU%3D&reserved=0)
- by asking one of the research team
- by sending an email to Kathy.liu@ucl.ac.uk, or
- by ringing Dr Liu on XXX

### **Appendix B. Clinical vignettes and scenarios.**

CMAI items and frequency scores in red and blue text were not shown in the survey.

| Scenario | Text | Scores |
| --- | --- | --- |
| Case 1 | Mary has Alzheimer’s disease dementia and needs help several times daily with personal care, including washing, getting dressed or undressed, and sometimes toileting. Staff at her residential home report that she always verbally refuses personal care, but can normally be distracted and reassured to accept it.  For the past month, Mary has shown increased distress when staff approach her for personal care. Once or twice a day **[5]**, when staff approach her for personal care, she screams **[#13]** and threatens that she will hit them **[#4]** if they touch her, and no longer reliably responds positively to their reassurance. At these times **[5]**, she also unsuccessfully tries to dress or undress herself while refusing assistance **[#2]**. Several times a week **[4]** during this period, she has pushed, scratched and kicked staff, **[#10, #15, #8]** and has hurt them.  Staff members have to spend considerably more time trying to offer her personal care than they used to. They describe her behaviour as very upsetting to her and to Staff and very disruptive, as they have less time to carry out activities that are usually devoted to other residents. | CMAI = 50  NPI A/A = 12 |
| 1A | After starting an intervention, *Mary’s kicking and pushing* **[#8, #10]** *at times of personal care has reduced in frequency from several times a week to once or twice per week* **[3]***.* She has still scratched and hurt staff several times a week during this period, as often as before. She still verbally refuses personal care several times a day, and screams, verbally threatens staff, and tries to unsuccessfully undress/dress herself once or twice a day as often as before. | CMAI = 48  NPI A/A = 12 |
| 1B | After continuing an intervention, Mary still refuses care several times a day, screams and threatens staff and tries unsuccessfully to dress/undress herself once or twice a day, as frequently as before. She has also kicked and pushed staff once or twice a week and has scratched and hurt staff several times a week, as before. *She is now more able to eventually respond to staff members' reassurance and can be distracted and accept personal care.* | CMAI = 48  NPI A/A = 8 |
| 1C | After continuing an intervention, Mary continues to verbally refuse personal care several times a day, accompanied by once to twice daily threats to hurt staff, and once or twice a week she has kicked and pushed staff and several times a week has scratched and hurt staff, as frequently as before. She is still able to eventually respond to staff reassurance and can be distracted and accept personal care, as before. *She now less frequently screams* **[#13]** *or attempts to unsuccessfully dress/undress herself* **[#2]** *(reduced from once or twice daily to several times per week* **[4]***) when approached by staff for personal care.* | CMAI = 46  NPI A/A = 8 |
| Case 2 | John has Alzheimer’s disease dementia and lives at home with his wife, Alice, who is his main caregiver. John needs to be accompanied if he leaves the house as he will become disorientated and get lost on his own, but does not demonstrate sufficient insight into this risk. Alice usually accompanies John for a walk outside once or twice a day. Several times an hour **[7]** during the day, John shows signs of restlessness **[#29]**. At these times **[7]**, he repeats sentences or questions **[#6]**, and paces around the house without an obvious purpose **[#1]**. Several times a day **[6]**, he will try to open the front door to go outside on his own **[#16]**. He cannot successfully open the door on his own and Alice can normally distract and reassure him to come away from the door.  For the past month, John appears more distressed when he tries to open the front door unsuccessfully. Once or twice a day **[5]**, when Alice tries to encourage him to come away from the door, he screams and swears at her **[#4, #13]**, and no longer reliably responds positively to her reassurance. Several times a week **[4]** during this period, John has banged and hit the door **[#7]**, which once or twice a week **[3]** has led to bruises and cuts **[#21]** appearing on his hand. Twice during this period **[2]**, he has thrown nearby furniture objects onto the ground **[#11]**, which has led to their breakage and damage **[#25]**.  Alice has to spend considerably more time trying to distract and reassure John than before and considers his behaviour to be very disruptive, as she feels she cannot devote time to other activities. She describes his behaviour as extremely distressing to John and herself. | CMAI = 67  NPI A/A = 12 |
| 2A | After starting an intervention, *John’s restlessness, repetitive sentences and questions and pacing and aimless wandering* **[#29, #6, #1]** *now occur several times a day* **[6]** *instead of several times an hour. This means he now tries to open the front door* **[#16]** *once or twice a day* **[5]** *instead of several times a day,* but he still screams and swears at Alice when she tries to reassure him once or twice a day, as often as before. He has also continued to bang or hit the door, which have hurt his hands, and occasionally damage furniture items by throwing them onto the ground, as often as before. | CMAI = 63  NPI A/A = 12 |
| 2B | After continuing an intervention, John has shown no change in symptoms, in that he still appears restless, says repetitive sentences and questions and paces aimlessly several times a day, and will scream and swear at Alice when he tries to open the front door once or twice a day, as often as before. He also continues to bang or hit the door and hurt his hands, and occasionally throw and damage furniture items, as often as before. *John is now more able to eventually respond to Alice's reassurance and be distracted to come away from the front door, which makes him less distressed.* | CMAI = 63  NPI A/A = 8 |
| 2C | After continuing an intervention, John continues to appear restless, says repetitive sentences and questions and paces aimlessly several times a day, and tries to open the front door once or twice a day, as often as before. Several times a week, he also continues to bang or hit the door and occasionally hurt his hands, and sometimes throws and damages furniture items, as often as before. He is still able to eventually respond to Alice’s reassurance and can be distracted and encouraged to walk away from the front door, as before. *He now less frequently screams and swears at Alice* **[#4, #13]** *at these times, which now occurs once or twice a week* **[3]** *instead of once or twice a day.* | CMAI = 59  NPI A/A = 8 |
| Case 3 | Harold has Alzheimer’s disease dementia and lives in a residential home where he receives personal care several times a day for washing and dressing. Several times an hour **[7]** during the day, when he encounters female residents or staff members, he will make verbal sexual advances **[#27]** and unwarranted requests of help or attention **[#5]**. Several times a day **[6]**, this includes him touching the other person and making physical sexual advances **[#28]**. Staff members are normally able to distract him very quickly.  For the past month, several times a day **[6]**, Harold has also inappropriately undressed himself in front of female staff or residents **[#2]**. At these times **[6]**, he has also grabbed onto them **[#9]** which has hurt them.  Staff have to spend considerably more time trying to distract and reassure Harold than before and they consider his behaviour to be difficult to redirect and very disruptive. They describe his behaviour as extremely distressing to staff and residents but report that Harold appears to experience little distress. | CMAI = 56  NPI A/A = 4 |
| 3A | After starting an intervention, *Harold no longer* **[1]** *makes verbal sexual advances* **[#27]** *to female residents or staff.* He still requests help or attention which appear to be unwarranted several times an hour, still makes physical sexual advances, inappropriately undresses himself, and grabs onto and hurts female residents and staff several times a day, as often as before. | CMAI = 50  NPI A/A = 4 |
| 3B | After continuing an intervention, Harold continues to no longer make verbal sexual advances to female residents or staff, and *now his apparent unwarranted requests of help or attention* **[#5]** *have reduced in frequency to several times a week* **[4]** *from several times an hour. Inappropriately undressing* **[#2]** *now occurs less than once a week* **[2]***, reduced from once or twice a day.* Several times a day, as often as before, he still makes physical sexual advances and grabs onto and hurts female residents and staff. | CMAI = 44  NPI A/A = 4 |
| 3C | After continuing an intervention, Harold continues to no longer make verbal sexual advances, and several times a week he makes unwarranted requests of help or attention to female residents or staff, as often as before. He inappropriately undresses himself less than once a week, as often as before. *He now less frequently makes physical sexual advances* **[#28]** *or grabs onto and hurts female residents and staff* **[#9]***, as these behaviours now occur several times a week* **[4]** *instead of several times a day.* | CMAI = 40  NPI A/A = 4 |

REFERENCES

[1] Brodaty H, Ames D, Snowdon J, Woodward M, Kirwan J, Clarnette R, et al. A randomized placebo-controlled trial of risperidone for the treatment of aggression, agitation, and psychosis of dementia. J Clin Psychiatry 2003;64:134–43.

[2] Banerjee S, High J, Stirling S, Shepstone L, Swart AM, Telling T, et al. Study of mirtazapine for agitated behaviours in dementia (SYMBAD): a randomised, double-blind, placebo-controlled trial. Lancet 2021;398:1487–97.

[3] Revicki D, Hays RD, Cella D, Sloan J. Recommended methods for determining responsiveness and minimally important differences for patient-reported outcomes. J Clin Epidemiol 2008;61:102–9.

[4] Lansdall CJ, McDougall F, Butler LM, Delmar P, Pross N, Qin S, et al. Establishing Clinically Meaningful Change on Outcome Assessments Frequently Used in Trials of Mild Cognitive Impairment Due to Alzheimer’s Disease. The Journal of Prevention of Alzheimer’s Disease 2022. https://doi.org/10.14283/jpad.2022.102.

[5] Webster L, Martin A, Livingston G. The minimum clinically important difference on the sleep disorders inventory for people with dementia. Int J Geriatr Psychiatry 2020;35:1418–23.
